# Mice with Heterozygous Deletion of Exon 3 in the *Gh* Gene Demonstrate Growth Retardation Caused by Reduced *Ghrhr* mRNA

**DOI:** 10.3390/ijms26031061

**Published:** 2025-01-26

**Authors:** Daisuke Ariyasu, Daisuke Higa, Ryo Tokudome, Takumi Yonemori, Hayate Shimada, Shinsuke Shibata, Kimi Araki

**Affiliations:** 1 Division of Developmental Genetics, Institute of Resource Development and Analysis, Kumamoto University, Honjo 860-0811, Kumamoto, Japan; daisukeariyasu@gmail.com (D.A.); touhutouhu003@gmail.com (D.H.); ryotokudome9@gmail.com (R.T.); 241y1029@st.kumamoto-u.ac.jp (T.Y.); greeeen-love96.06_06@outlook.jp (H.S.); 2 Department of Pediatrics, Kawasaki Municipal Hospital, Kawasaki 210-0013, Kanagawa, Japan; 3 Division of Microscopic Anatomy, Graduate School of Medical and Dental Sciences, Niigata University, 1-757 Asahimachi-Dori, Chuo-Ku, Niigata 951-8510, Niigata, Japan; shibatas@med.niigata-u.ac.jp; 4 Center for Metabolic Regulation of Healthy Aging, Kumamoto University, 1-1-1, Honjo 860-8556, Kumamoto, Japan

**Keywords:** isolated growth hormone deficiency type 2, endoplasmic reticulum stress, growth hormone releasing hormone receptor, transcriptome analysis

## Abstract

Isolated Growth Hormone Deficiency Type 2 (IGHD2) is caused by a heterozygous splice site variant in intron 3 of the *GH1* gene. The resulting exon 3-skipped growth hormone (Δ3 GH), produced from the mutated allele, exerts a dominant-negative effect, leading to growth hormone (GH) deficiency. However, the precise molecular mechanisms underlying this effect remain poorly understood. While several model murine models expressing human Δ3 GH have been developed, no IGHD2 mouse models featuring variants in the endogenous *Gh* gene currently exist. We generated a mouse model (*Gh*^+/Δ3^) with a heterozygous deletion of exon 3 in the *Gh* gene using CRISPR/Cas9 system. The *Gh*^+/Δ3^ model exhibited GH deficiency caused by a dominant-negative effect at the mRNA level, characterized by reduced *Gh* mRNA expression. This mechanism parallels findings in our previous humanized IGHD2 mouse model, where the deficiency was driven by decreased *Ghrhr* mRNA expression. Transcriptome analysis of the pituitary revealed widespread downregulation of mRNAs encoding membrane and secretory proteins. The dominant-negative effect of Δ3 GH in IGHD2 is mediated by properties of Δ3 GH that are conserved across both humans and mice. This mechanism involves the downregulation of mRNAs, including those encoding membrane and secretory proteins, such as *Ghrhr* mRNA.

## 1. Introduction

Isolated Growth Hormone Deficiency Type 2 (IGHD2) is primarily caused by a heterozygous splice-site variant in exon 3 of the *GH1* gene. This variant leads to exon 3 skipping during splicing, resulting in the pathological overproduction of a 17.5 kDa isoform of human growth hormone (Δ3 hGH), which is normally expressed at minimal levels in healthy pituitary glands [1]. Despite one allele being wild-type, patients with IGHD2 exhibit growth hormone (GH) deficiency, suggesting that Δ3 hGH exerts a dominant-negative effect. However, the molecular mechanisms underlying this effect remain incompletely understood, 30 years after IGHD2 was first reported in 1994 [1]. Δ3 hGH lacks the loop structure formed by amino acids 32–71, which normally connects the first and second α-helices. This structural alteration disrupts proper protein folding within the endoplasmic reticulum (ER) [2]. Consequently, Δ3 hGH is retained in the ER rather than progressing through the secretory pathway to the Golgi apparatus. It is ultimately degraded in the cytoplasm via ER-associated degradation (ERAD) and is not secreted into the bloodstream [3,4].

A notable feature of Δ3 hGH is the presence of cysteine residue (p.Cys53) in the deleted loop. Previous studies have proposed that Δ3 hGH may enable the formation of intermolecular heterodimers with wild-type (WT) hGH via disulfide bonds involving free cysteine residues (Cys165) in Δ3 hGH. However, evidence supporting heterodimer formation between WT hGH and Δ3 hGH is lacking. Furthermore, in families with a missense variant (p.Cys53Ser) in exon 3 of the *GH1* gene, only homozygotes exhibit short stature, suggesting no dominant-negative effect in these cases [5]. While the dominant-negative effect in IGHD2 is widely hypothesized to result from protein interactions between WT hGH and Δ3 hGH rather than inherent toxicity of Δ3 hGH [2,6,7], some evidence suggests that Δ3 hGH alone may induce ER stress, potentially leading to apoptosis [4].

Due to the limited availability of pituitary samples from IGHD2 patients, most insights into these mechanisms have been derived from in vitro studies conducted during the decade following the initial report of the disorder. These studies employed various methods to assess dominant-negative effects, including transient overexpression of wild-type and mutant constructs in pituitary-derived cell lines to quantify WT hGH secretion [6,8], stable expression of mutant constructs [9], and inducible expression of mutant constructs in cells with stable WT construct expression [4]. Most of these studies operated under the assumption that the dominant-negative effect occurs at the protein level, which was verified by manipulating the amount of mutant DNA for transformation under the presence of constant WT hGH expression.

However, in vivo GH secretion in the pituitary is regulated transcriptionally by hypothalamic growth hormone-releasing hormone (GHRH) and somatostatin in response to insulin-like growth factor 1 (IGF-1) feedback. Thus, animal models are essential for a precise understanding of the dominant-negative effects of Δ3 hGH in vivo. In 2003, the first IGHD2 animal model, a transgenic mouse expressing Δ3 hGH, was developed. These mice exhibited growth impairment consistent with the IGHD2 phenotype. However, severe overexpression of Δ3 hGH caused extensive inflammation and destruction of the pituitary, precluding a detailed investigation into the molecular effects of Δ3 hGH on GH-producing cells [10]. In 2019, our group generated a “humanized IGHD2 model mouse” designed to accurately mimic the IGHD2 phenotype. This model was generated by sequentially replacing the two alleles of the murine *Gh* gene with a single copy of the wild-type human *GH1* gene (*WThGH1*) and a single copy of a mutant human *GH1* gene (*Δ3hGH1*) containing an intron 3 donor site (IVS3 ds) variant. In this model, GH secretion deficiency was associated with decreased *GH1* gene expression at the mRNA level in the pituitary, compared to healthy control mice homozygous for *WThGH1.* Importantly, no evidence of cell death was observed. This reduction in *GH1* mRNA was attributed to decreased *Ghrhr* mRNA expression, which encodes the GHRH receptor, and was evident even in mice carrying only the mutant allele (without the wild-type allele). These findings demonstrated that the dominant-negative effect of Δ3 hGH in the humanized IGHD2 mice model does not result from protein–protein interactions between WT hGH and Δ3 hGH [11].

Based on these observations, we hypothesized that the dominant-negative effect in IGHD2 arises from the intrinsic properties of Δ3 hGH, which independently affect somatotroph function. To investigate whether these properties are unique to human Δ3 hGH or conserved across species, we generated a mouse model producing a murine GH mutant (Δ3 mGH) by deleting exon 3 of the *Gh* gene at the genomic level. Using transcriptome analysis of the pituitary, we sought to elucidate the molecular basis of the dominant-negative effect. In this study, we report that Δ3 mGH exerts a dominant-negative effect on wild-type *Gh* alleles in mice, similar to the IGHD2 phenotype. This study provides new insights into the pathophysiology of IGHD2.

## 2. Results

### 2.1. Mice with a Heterozygous Deletion of Exon 3 of the Gh Gene Exhibit Moderate Growth Retardation

**Figure 1:**
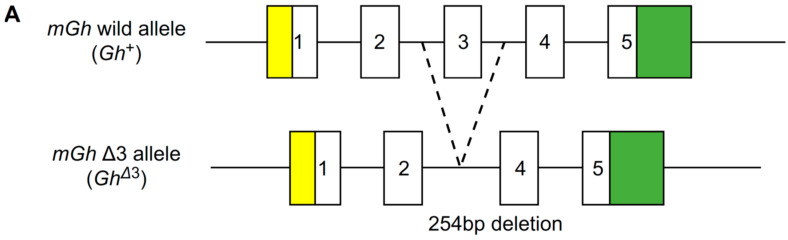

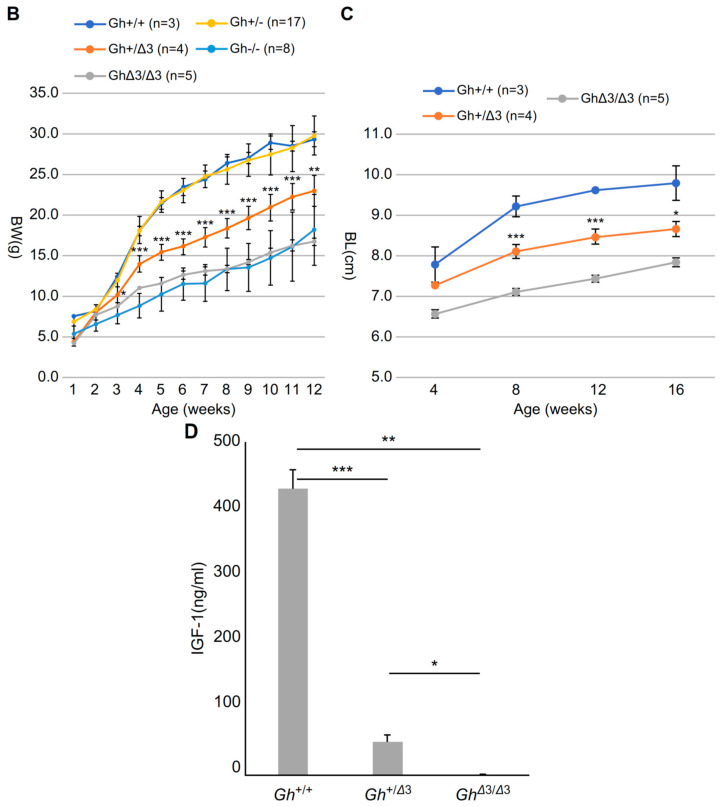
(**A**) Schematic representation of the *mGh* wild allele (*Gh*^+^) and edited allele (*Gh*^Δ3^). Open rectangles represent the open reading frames of the *Gh* gene. Yellow and green rectangles represent untranslated regions of the *Gh* gene. The numbers within rectangles indicate exons of the *Gh* gene. (**B**) Body weight of male *Gh*^+/+^, *Gh*^+/Δ3^, *Gh*^Δ3/Δ3^, *Gh*^+/^*^−^*, and *Gh^−^*^/*−*^ mice. Data represent the mean ± SD. * *p* < 0.05, ** *p* < 0.1, *** *p* < 0.005. (**C**) Body lengths of male *Gh*^+/+^, *Gh*^+/Δ3^, and *Gh*^Δ3/Δ3^ mice. Data represent the mean ± SD. * *p* < 0.05, *** *p* < 0.005. (**D**) Serum IGF-1 values in 4-week-old model mice. Data represent the mean ± SD, n = 3. * *p* < 0.05, ** *p* < 0.01, *** *p* < 0.005.

A schematic representation of the wild-type allele and the mutant allele generated using the CRISPR/Cas9 system is shown in Figure 1A. Growth curves of body weight and length for wild-type (*Gh*^+/+^), IGHD2 mice model (*Gh*^+/Δ3^), homozygous Δ3 allele mice (*Gh*^Δ3/Δ3^), heterozygous Gh deletion mice from a previous study using homologous recombination (*Gh*^+/*−*^), and Gh KO mice (*Gh^−^*^/*−*^) are shown in Figure 1B,C. *Gh^−^*^/*−*^ mice demonstrated severe postnatal growth retardation, indicating that, as in humans, postnatal growth in mice is GH-dependent. *Gh*^+/Δ3^ mice exhibited moderate growth retardation, consistent with the IGHD2 phenotype, and were significantly smaller than *Gh*^+/*-*^ mice but larger than *Gh^−^*^/*−*^ mice. No transcripts other than exon 3-skipped *Gh* transcripts were produced from the genome-edited *Gh*^Δ3^ allele. *Gh*^Δ3/Δ3^ mice displayed growth impairment consistent to that of *Gh^−^*^/*−*^ mice, aligning with in vitro data showing that Δ3 GH is not secreted extracellularly. IGF-1 levels in *Gh*^+/Δ3^ mice were also markedly lower than those in wild-type, suggesting that the observed phenotype is due to GH deficiency (Figure 1D).

### 2.2. GH Deficiency in Gh^+/Δ3^ Mice Is Caused by a Decrease in Ghrhr mRNA

**Figure 2:**
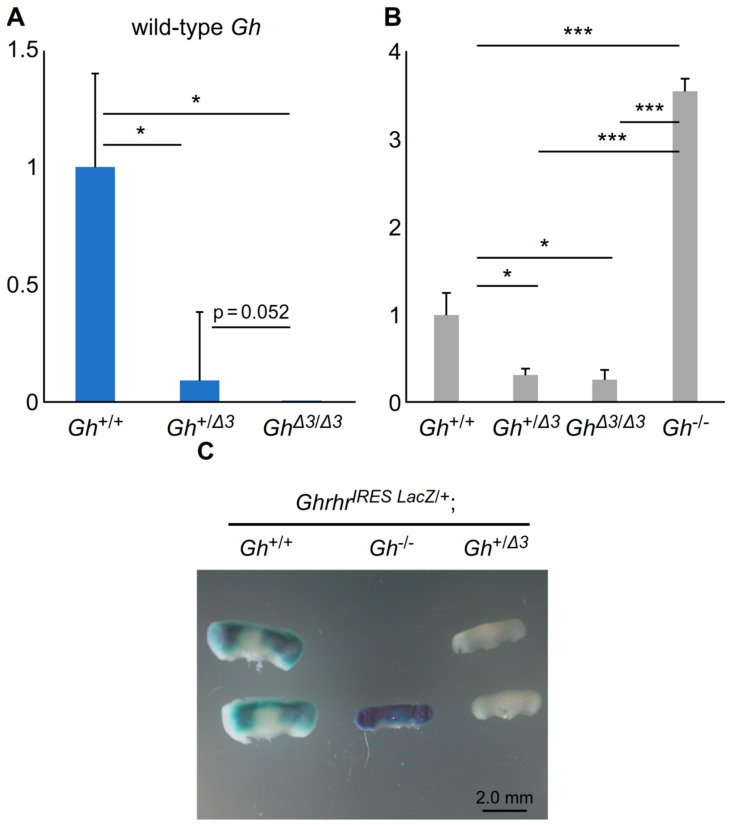
qRT-PCR analysis of (**A**) wild-type *Gh* and (**B**) *Ghrhr* mRNA levels using pituitary samples from model mice at 4 weeks of age. (**C**) Results of X-gal staining of pituitary glands from 4-week-old *Ghrhr*^+/*IRES-LacZ*^; *Gh*^+/+^, *Ghrhr*^+/*IRES-LacZ*^*; Gh*^+/Δ3^, and *Ghrhr*^+/*IRES-LacZ*^*;Gh^−^*^/*−*^ mice. * *p* < 0.05, *** *p* < 0.005. Scale bar, 2 mm.

Our previous study demonstrated that GH deficiency in humanized IGHD2 model mice was caused by reduced GH1 mRNA, mediated by a decrease in *Ghrhr* mRNA levels. To determine whether a similar mechanism occurs in *Gh*^+/Δ3^ mice, the transcriptional products of the wild-type *Gh* gene were examined using qRT-PCR. qRT-PCR analysis using a sense primer in exon 3 and an antisense primer spanning exons 3 and 4, specifically targeting wild-type *Gh* mRNA, revealed a significant reduction in wild-type *Gh* mRNA in *Gh*^+/Δ3^ levels compared to *Gh*^+/+^. The levels were less than half of the normal amount, indicating that, similar to previous findings, the dominant-negative effect was observed at the mRNA level (Figure 2A). Subsequently, *Ghrhr* mRNA levels were assessed by qRT-PCR. In *Gh^−^*^/*−*^ mice, which completely lack GH, an increase in *Ghrhr* mRNA levels due to negative feedback was observed. However, despite the apparent GH-deficient state in *Gh*^+/Δ3^ mice, *Ghrhr* mRNA levels were significantly lower than those in *Gh*^+/+^ mice. This finding strongly suggests that the reduction in *Gh* mRNA levels in *Gh*^+/Δ3^ mice may be mediated by decreased *Ghrhr* mRNA expression. Furthermore, a notable difference in *Ghrhr* mRNA expression levels was observed between *Gh^−^*^/*−*^ and *Gh*^Δ3/Δ3^ mice, both of which are expected to exhibit complete GH deficiency. This observation confirmed that the dominant-negative effect is fundamentally caused by a reduction in *Ghrhr* mRNA levels. Moreover, the decrease in *Ghrhr* mRNA mediated by Δ3 mGH is independent of WT mGH (Figure 2B).

In addition, mice expressing *LacZ* mRNA from the *Ghrhr* gene locus via an IRES sequence were generated. X-gal staining was performed on *Gh*^+/Δ3^ mice crossed with these LacZ reporter mice to evaluate the promoter activity of the *Ghrhr* gene. The results showed a marked decrease in X-gal staining in *Gh*^+/Δ3^ mice. This strongly suggests that, similar to the humanized IGHD2 model, the promoter activity of the *Ghrhr* gene in *Gh*^+/Δ3^ is reduced via a similar mechanism (Figure 2C).

### 2.3. Significant ER Proliferation and Reduced Secretory Granules in Somatotrophs of Gh^+/Δ3^ Mice, with Mild ER Stress

**Figure 3:**
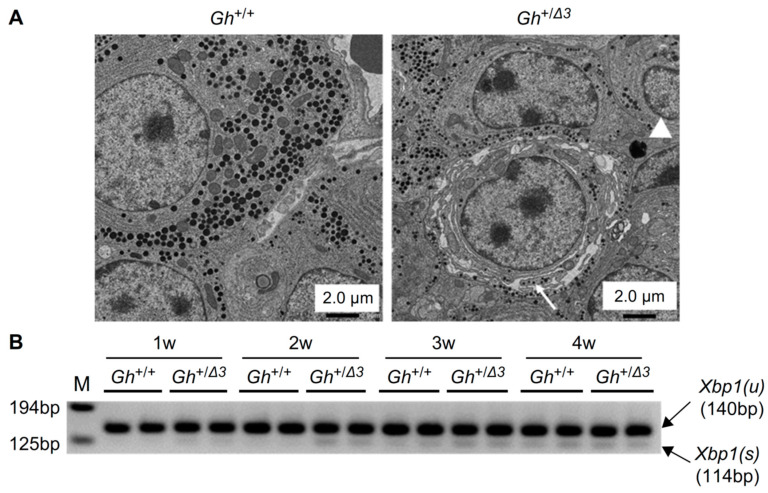

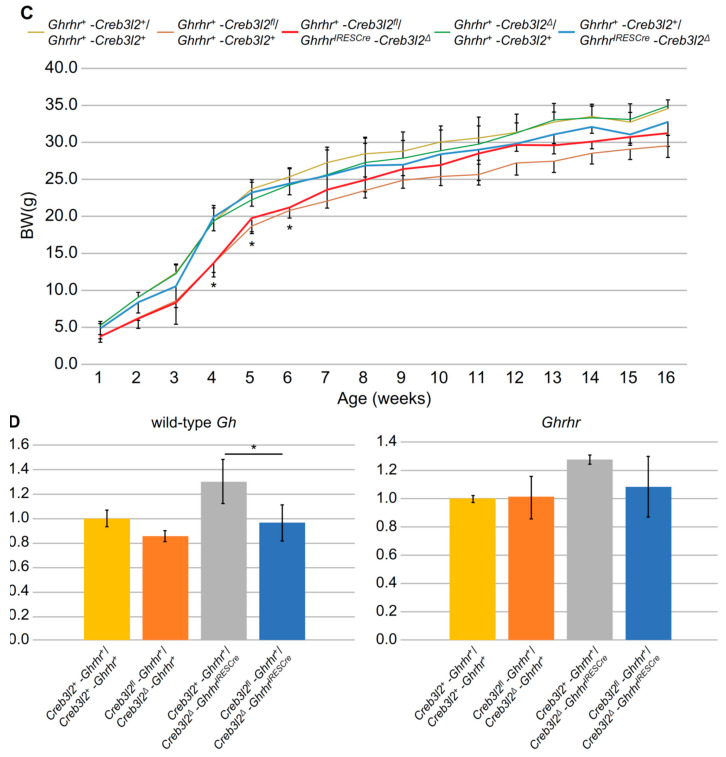
(**A**) TEM images of pituitary glands from 4-week-old *Gh*^+/+^, and *Gh*^+/Δ3^ mice. Marked enlargement of the rough endoplasmic reticulum (arrow) and cytosolic black aggregates (arrowhead) were observed in *Gh*^+/Δ3^ somatotrophs. Scale bars, 2 µm. (**B**) RT-PCR of *Xbp1* mRNA splicing. Primers surrounding the region spliced out by IRE1 were used. PCR products of 140 and 114 bp represent unspliced [*Xbp1(u)*] and spliced [*Xbp1(s)*] Xbp1, respectively. (**C**). Body weights of male wild-type (yellow), *Creb3l2^fl^*^/+^ (brown), *Ghrhr*^+/*IRESCre*^*;Creb3l2^fl^*^/Δ^ (red, *Creb3l2* conditional KO), *Creb3l2*^Δ/+^ (green), and *Ghrhr*^+/*IRESCre*^*;Creb3l2*^+/Δ^ mice (blue). Please note that the conditional KO group (red) did not exhibit significant differences in body weight compared to the control group (blue), except at certain time points. Data represent the mean ± SD. * *p* < 0.05. (**D**). qRT-PCR analysis of wild-type *Gh* and *Ghrhr* mRNA levels using pituitary samples from model mice at 4 weeks of age. * *p* < 0.05.

To evaluate the effects of Δ3 mGH on somatotrophs, electron microscopy imaging of the pituitary glands in 4-week-old mice was conducted. In the pituitaries of *Gh*^+/+^ mice, numerous secretory granules and a moderately developed ER were observed. In contrast, *Gh*^+/Δ3^ mice displayed marked expansion of the ER, a reduction in secretory granules, and high electron density protein aggregates within the cytoplasm contiguous with the ER membrane (Figure 3A). These findings suggest that, similar to Δ3 hGH, Δ3 mGH localizes to the ER and may subsequently undergo degradation in the cytoplasm. Additionally, the accumulation of Δ3 mGH in the somatotroph ER may induce ER stress.

Abnormal proteins accumulated in the ER are retrotranslocated to the cytoplasm and degraded through ERAD via the ubiquitin-proteasome pathway [12]. Misfolded proteins in the ER are detected by the ER stress sensor IRE1. IRE1, functioning as an RNase, initiates splicing of *Xbp1* mRNA in the cytoplasm. The spliced *Xbp1* mRNA is then translated into the active transcription factor XBP1(S), which induces the production of ERAD components [13]. Thus, the splicing of *Xbp1* mRNA was assessed via competitive RT-PCR. In *Gh*^+/Δ3^ mice, *Xbp1* mRNA splicing was slightly increased compared to *Gh*^+/+^ mice. However, this level of ER stress was not severe enough to induce apoptosis. Therefore, while Δ3 mGH does induce ER stress, it is unlikely that cell death from ER stress is the cause of the observed reduction in *Ghrhr* mRNA (Figure 3B).

### 2.4. Reduction of Nuclear CREB3L2 Is Not the Cause of the Dominant-Negative Effect in IGHD2

In our previous study, the Creb3 family, which localizes to the ER and, upon cleavage of its N-terminus, acts as a nuclear transcription factor within the nucleus was focused and studied [14]. In the humanized IGHD2 model mice, nuclear CREB3L2 levels were reduced and CREB3L2 was identified as a novel nuclear transcription factor that binds to the promoter region of the *Ghrhr* gene. This indicated that Δ3 hGH could potentially inhibit *Ghrhr* mRNA transcription via a reduction in nuclear CREB3L2 protein, which might contribute to the dominant-negative effect observed in this condition. If this hypothesis were correct, then *Creb3l2* KO mice should also exhibit similar growth defects due to a reduction in *Ghrhr* mRNA levels. Since *Creb3l2* KO mice die shortly after birth [15], somatotroph-specific *Creb3l2* KO mice was created for the purpose of investigating this phenomenon in vivo (Supplementary Figures S1 and S2A,B). The results showed that the somatotroph-specific *Creb3l2* KO mice did not exhibit growth defects (Figure 3C), and neither *Ghrhr* mRNA nor *Gh* mRNA levels were reduced (Figure 3D). These findings confirm that a reduction in nuclear CREB3L2 is not the cause of the dominant-negative effect in this condition.

### 2.5. Δ3 mGH Causes a Generalized Reduction in Transcripts of Membrane and Secretory Proteins in Pituitary Somatotrophs

Based on the findings described above, Δ3 mGH was shown to induce a decrease in *Ghrhr* mRNA, which in turn leads to reduced GHRH signaling and subsequent reduction of *Gh* mRNA. Additionally, Δ3 mGH was found to cause abnormalities in intracellular organelles, such as ER expansion and a decrease in secretory granules. However, the precise mechanism behind the decrease in *Ghrhr* mRNA remained unclear.

**Figure 4:**
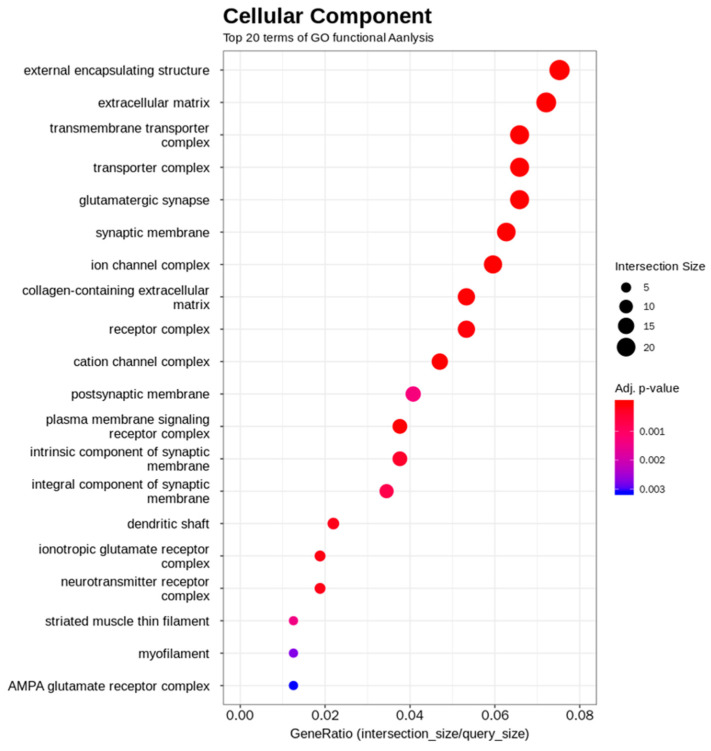
The results of the ontology analysis of genes decreased in *Gh^−^*^/Δ3^ compared with *Gh^−^*^/*−*^. Numerous mRNAs encoding membrane proteins, such as receptors, channels, and transporters, as well as secreted proteins, including extracellular matrix components and hormones, were markedly reduced.

For the purpose of exploring this further, a transcriptome analysis using pituitary tissue was performed. Since previous findings confirmed that the decrease in *Ghrhr* mRNA induced by Δ3 mGH does not require the presence of the wild-type *Gh* allele, transcriptome analysis on the pituitaries of *Gh^−^*^/*−*^ and *Gh^−^*^/Δ3^ mice was performed. Enrichment analysis using GO and pathway annotations revealed a widespread and significant reduction in transcripts related to membrane proteins, such as receptors, channels, and transporters, as well as in transcripts of secretory proteins, including GH and extracellular matrix components (Figure 4).

## 3. Discussion

In the humanized IGHD2 model mice generated in 2019, an unexpected phenomenon was observed: a dominant-negative effect at the mRNA level, where the mRNA of the substituted *hGH1* gene itself was reduced. This unique, previously undocumented occurrence demonstrated that the reduction in *hGH1* mRNA was due to a decrease in *Ghrhr* mRNA. However, it was difficult to completely rule out the possibility that the replacement of the endogenous *Gh* gene with the *hGH1* gene partially influenced this effect by secondarily reducing *Gh* promoter activity. In the current study, we confirmed the exact same phenomenon in mice in which the *Gh* gene was genome-edited to delete exon 3 on one allele. This finding strongly suggests that the dominant-negative effect observed at the mRNA level in our prior research was not a secondary effect of gene manipulation when substituting the mouse’s endogenous gene but rather is due to an intrinsic characteristic of Δ3 hGH and Δ3 mGH proteins. This suggests the existence of a conserved mechanism, independent of species, driven by the shared properties of these Δ3 GH proteins.

This study, along with our previous research, has demonstrated that an increase in the production of GH lacking exon 3 in pituitary somatotrophs exerts a dominant-negative effect on the wild-type allele, primarily by reducing *Ghrhr* mRNA levels. The marked ER expansion observed in somatotrophs of both humanized IGHD2 model mice and *Gh*^+/Δ3^ mice suggest that somatotrophs are subjected to ER stress. However, unlike typical endocrine diseases caused by ER stress, where apoptosis and cell death significantly contribute to pathology, no cell death was observed in the humanized IGHD2 model mice. Additionally, both previous and current studies show only mild activation of the classical ER stress response in these model mice (Figure 3B) [11]. Based on these findings, it is likely that the reduction in *Ghrhr* mRNA is driven by a mechanism distinct from the ER stress response. The ER expansion appears to be a result of Δ3 GH accumulation in the ER, rather than a direct cause of the reduction in *Ghrhr* mRNA.

To investigate the mechanism behind the reduction in *Ghrhr* mRNA, a transcriptome analysis of mouse pituitary glands was performed. Since it was confirmed that the presence of the wild-type allele does not affect the reduction in *Ghrhr* mRNA, transcriptome analysis was performed on *Gh^−^*^/*−*^ and *Gh^−^*^/Δ3^ mice. This approach allowed us to exclude any effects related to haploinsufficiency of the wild-type *Gh* allele and to assess the specific impact of the *Gh*^Δ3^ allele on intracellular transcript levels. The analysis revealed a comprehensive reduction in the transcripts of membrane and secretory proteins. Notably, the reduction was observed not only in transcripts for proteins directly related to GH secretion, such as *Ghrhr*, *Ghsr*, and *Sstr5*, but also in those of membrane and secretory proteins unrelated to GH secretion, including ion channels and extracellular matrix components. Given that most membrane and secretory proteins are translated in the ER, this phenomenon likely reduces the protein load on the ER, thereby protecting the cell from ER stress and potentially helping to prevent cell death.

If the broad reduction in mRNA levels results from the decreased transcription of each gene, the most plausible explanation is that Δ3 GH-induced ER stress weakens somatotroph’s entire activity. In both our previous and current studies, mice that express β-galactosidase from the *Ghrhr* gene locus were used for X-gal staining on the pituitary gland, demonstrating a drastic reduction in staining in this disease model. This *LacZ* knock-in mouse is widely used as an in vivo tool to evaluate gene promoter activity, indicating that Δ3 GH somehow reduces the promoter activity of the *Ghrhr* gene. This suggests that Δ3 GH-induced ER stress leads to a general decline in somatotroph function.

Apart from the reduced somatotroph activity due to ER stress, another possible mechanism for the decline in *Ghrhr* gene promoter activity could be the decrease in nuclear transcription factors that bind upstream of the *Ghrhr* gene. In previous studies, CREB3L2 was first demonstrated in vitro to bind upstream of the *Ghrhr* gene. These findings led us to generate somatotroph-specific *Creb3l2* KO mice in this study, but they did not replicate the phenotype observed in this condition. Furthermore, a selective KO in *Creb3l2* alone cannot explain the widespread mRNA reduction observed, leading us to conclude that it is unlikely that the CREB3 family plays a role in the pathogenesis of this condition.

If the widespread mRNA reduction is attributed to mRNA degradation, one possible mechanism could involve regulated IRE1-dependent decay of mRNA (RIDD). Among the three known ER stress sensors—PERK, ATF6, and IRE1—IRE1 has RNase activity, and RIDD is a well-documented ER stress response mediated by IRE1. RIDD works by degrading mRNAs located near the ER, thereby reducing the protein load on the ER by limiting the translation of these mRNAs in the ER vicinity [16,17]. If it is hypothesized that Δ3 GH localized in the ER activates RIDD, it would be consistent with the observed reduction in mRNAs coding for membrane and secretory proteins, as these mRNAs, located near the ER, would be degraded. However, for mRNA to be a target of RIDD, a specific stem-loop structure in the mRNA is required, which does not explain the comprehensive reduction of a wide range of mRNAs [16]. In recent years, a novel ER stress pathway, known as ER stress-associated RNA silencing (ERAS), has been proposed. This pathway reduces the mRNA influx into the ER and the amount of nascent protein translated by degrading mRNA located near the ER through a mechanism similar to RNA interference, thereby alleviating protein load on the ER [18]. The existence of ERAS has been demonstrated in organisms ranging from nematodes to mammals. If ERAS is involved in the dominant-negative effect observed in this condition, a comprehensive reduction in mRNAs of ion channels, transporters, and other molecules, which are seemingly unrelated to GH secretion, could provide a unified explanation. Further research is warranted.

This study has several limitations. First, we were unable to demonstrate the localization of Δ3 mGH to the ER. Immunostaining could not be performed because antibodies that recognize WT mGH do not recognize Δ3 mGH, and no antibody specific to Δ3 mGH is available. Establishing a mouse model in which a tag sequence is attached to the C-terminus of Δ3 mGH may allow future immunostaining using anti-tag antibodies to confirm the localization of Δ3 mGH. Second, we did not obtain evidence of activation of ER stress responses other than the IRE1-Xbp1 pathway in the somatotrophs of *Gh*^+/Δ3^ mice. Third, we were unable to investigate the presence or absence of apoptosis in the *Gh*^+/Δ3^ mice in this study. As with the humanized IGHD2 model mice used in our previous study, it is speculated that in *Gh*^+/Δ3^ mice, ER stress is mildly activated and cell death remains negative. However, further research is required to confirm this.This study revealed that Δ3 mGH induces a broad reduction in mRNAs for membrane and secretory proteins in mouse somatotrophs, including a decrease in *Ghrhr* mRNA, which contributes to the onset of GH secretion deficiency. The detailed mechanism behind this widespread mRNA reduction remains unclear and requires further investigation.

## 4. Materials and Methods

### 4.1. Establishment of the Gh^+/Δ3^ Mice

Using the CRISPR-Cas9 system, we generated a total of 176 pronuclear-stage embryos (C57BL6/N mice), obtained via in vitro fertilization. The embryos were electroporated using a Super Electroporator NEPA 21 (NEPAGENE, Ichikawa, Japan) with the following components: Oligonucleotides, including two crRNAs (5 µM each), tracrRNA (10 µM), and single-stranded oligo DNA (ssODN, 1 µg/µL) for homologous recombination. The sequences (PAM sequences in parentheses) are as follows: crRNA for cutting intron 2: AGTCATTGAGGGAAACTATG(GGG), crRNA for cutting intron 3: GGGCTGCATGGAGGGAAACG(AGG), ssODN: TGCAGTTAGGGCTGCATGGAGGGAAttcAGTTTCCCTCAATGACTTCCTGGGG. The lowercase “ttc” indicates a three-base insertion at the cutting site, distinguishing non-homologous end joining from homologous recombination outcomes. crRNA and tracrRNA were sourced from Fasmac (Atsugi, Japan). The embryos were cultured overnight, and those that developed to the two-cell stage were transferred into surrogate mothers. Genomic DNA from 35 resulting pups was analyzed via polymerase chain reaction (PCR) and direct sequencing to confirm the deletion of 241 bp, including exon 3. Mice with the desired deletion were selected and backcrossed with C57BL6/N mice to establish the line.

### 4.2. Establishment of Somatotroph-Specific Creb3l2 Conditional Knockout (KO) Mice

A Cre driver mouse was developed by inserting the *Cre* sequence downstream of the *Ghrhr* gene using an internal ribosome entry site (IRES) (Supplementary Figure S1). In ES cells derived from C57BL/6 mice, an *IRES-NLSCre* construct, flanked by 5′ and 3′ homology arms for *Ghrhr*, was electroporated along with a Cas9 construct carrying the D10A variant [19,20]. ES clones with successful homologous recombination were identified via Southern blot analysis. An F0 chimera with 100% chimerism was crossed with C57BL6/N mice to generate N1 offspring.

*Creb3l2* floxed mice were generated by electroporating 200 fertilized C57BL/6N mouse embryos, obtained via in vitro fertilization, with the CRISPR-Cas9 system. This approach induced double-strand breaks in introns 3 and 4 of the *Creb3l2* gene, flanking exon 4, and simultaneously inserting *lox*P-containing oligonucleotides at these sites. After transferring the embryos to surrogate mothers, 13 offspring were obtained, one of which successfully carried *lox*P sequences in both introns 3 and 4. All N2 offspring inheriting the *lox*P sequences from this N1 individual retained both *lox*P sites, confirming that the *lox*P sequences were inserted in cis. Since the *Ghrhr* gene is expressed in the testes, male *Ghrhr^IRESCre^; Creb3l2^flox^* mice occasionally produced sperm lacking exon 4 of the *Creb3l2* gene (*Creb3l2*^Δ^). This could result in progeny inheriting a systemic KO of *Creb3l2* gene. To prevent this, we aimed to generate *Creb3l2^fl^*^/Δ^*; Ghrhr*^+/*IRESCre*^ mice, ensuring that the *Creb3l2^fl^* allele was inherited from the female and both the *Creb3l2*^Δ^ and *Ghrhr^IRESCre^* alleles were inherited from the male (Supplementary Figure S2A). Furthermore, the *Ghrhr* and *Creb3l2* genes are located on the same chromosome, separated by a genetic distance of 10 cM. To generate individual mice carrying *Ghrhr^IRES-Cre^;Creb3l2^fl^* alleles in cis, artificial insemination was performed, mating *Creb3l2*^+/*fl*^; *Ghrhr*^+/+^ males with *Creb3l2*^+/*fl*^*; Ghrhr^IRESCre^*^/+^ females. This yielded *Creb3l2^fl^*^/*fl*^*;Ghrhr*^+/*IRESCre*^ mice at a frequency of approximately 2.5% (Supplementary Figure S2B).

### 4.3. Establishment of Somatotroph-Specific LacZ-Expressing Mice

To evaluate the population of pituitary somatotrophs and assess *Ghrhr* promoter activity, we established mice expressing NLS-LacZ downstream of the *Ghrhr* gene using IRES. This was achieved using the same D10A-Cas9-based method described for the Cre driver mice (Supplementary Figure S1).

### 4.4. RT-PCR and Quantitative RT-PCR

Total RNA was extracted from mouse pituitaries using the RNeasy mini kit (Qiagen, Hilden, Germany). From each sample, 250 ng of RNA was transcribed into cDNA using ReverTra Ace (FSQ-101; Toyobo, Osaka, Japan). Aliquots of cDNA were then analyzed using RT-PCR and quantitative RT-PCR (qRT-PCR). For qRT-PCR, reactions were performed with Thunderbird SYBR quantitative PCR mix (QPS-201; Toyobo) as Taq polymerase on an Applied Biosystems 7500 real-time PCR system (Applied Biosystems, Foster City, CA, USA) using the SYBR method. Relative mRNA expression levels were calculated using a standard curve and normalized to β-actin expression in experiments using relative quantification.

### 4.5. Transmission Electron Microscopy (TEM)

Pituitary glands from 4-week-old mice were prepared for TEM as previously described [21]. Tissues were fixed in 2.5% glutaraldehyde in 0.1 M phosphate buffer (pH 7.4) at 4 °C for 24 h, followed by a 2-h postfixation in 1% osmium tetroxide. Samples were dehydrated through graded ethanol and acetone solutions with n-butyl glycidyl ether (QY1), including a graded concentration of Epon with QY1. The tissues were then embedded into 100% Epon. Following 72-h polymerization in pure Epon, 70-nm ultrathin sections of the pituitary glands were mounted on copper grids, stained with uranyl acetate and lead citrate for 10 min, and visualized using a TEM (JEM-1400 plus; Jeol, Tokyo, Japan).

### 4.6. X-Gal Staining

Pituitary glands from 4-week-old mice were fixed in 4% paraformaldehyde for 1 h on ice. After fixation, tissues were permeabilized in rinse buffer (PBS with 2 mM MgCl_2_, 0.01% sodium deoxycholate, and 0.02% Nonidet P-40) for 2 h on ice, followed by three 30-min washes in PBS. Samples were stained overnight at 37 °C in rinse buffer containing 5 mM potassium ferricyanide, 5 mM potassium ferrocyanide, and 1 mg/mL 5-bromo-4-chloro-3-indolyl-b-D-galactoside (X-gal).

### 4.7. Transcriptome Analysis

For transcriptome analysis, pituitary tissues were collected from three 2-week-old male mice of each genotype (*Gh*^Δ3/*−*^ and *Gh^−^*^/*−*^, respectively). The mice were euthanized by decapitation, and total RNA was extracted from their pituitaries using the RNeasy Mini Kit (Qiagen, Hilden, Germany). Of the 45,777 genes detected across all samples, 24,955 genes with undetectable expression in at least one sample were excluded, leaving 20,822 genes for further analysis. TMM normalization was applied, followed by transcriptome analysis. For genes showing a two-fold or greater difference in expression, enrichment analysis was performed to identify gene ontology (GO) and pathway annotations using FastQC v0.11.7, Trimmomatic 0.38, HISAT2 version 2.1.0, and StringTie version 2.1.3b. The transcriptome analysis was outsourced to Macrogen Japan Corporation (Tokyo, Japan).

### 4.8. Statistical Analysis

Data were analyzed using a two-tailed unpaired Student’s *t*-test for comparisons between the two groups. Results are presented as means ± SD.

### 4.9. Study Approval

The Animal Care Committee and Institutional Biosafety Committee of Kumamoto University approved all mouse protocols. All experiments were performed in accordance with the Declaration of Helsinki and approved by the Kumamoto University Ethics Committee for Animal Experiments (authorization numbers: A2019-108R2, A2021-035R1, and A2023-054R3).

## 5. Conclusions

The IGHD2 model mice, in which exon 3 of the *Gh* gene was deleted via genome editing, exhibited a dominant-negative effect at the mRNA level. This was characterized by a reduction in *Gh* mRNA resulting from decreased *Ghrhr* mRNA, mediated through a comprehensive reduction in transcripts encoding membrane and secretory proteins.

## Data Availability

Data is contained within the article and Supplementary Materials.

## Supplementary Materials

The following supporting information can be downloaded at: https://www.mdpi.com/article/10.3390/ijms26031061/s1.
