# Supplementary material for: Mice with Heterozygous Deletion of Exon 3 in the Gh Gene Demonstrate Growth Retardation Caused by Reduced Ghrhr mRNA
Source: Int J Mol Sci. 2025 Jan 26;26(3):1061. doi: 10.3390/ijms26031061 (PMC11817076; doi:10.3390/ijms26031061)
Supplement: Supplementary file 1 [file ijms-26-01061-s001.zip › ijms-3367545-supplementary.pdf]

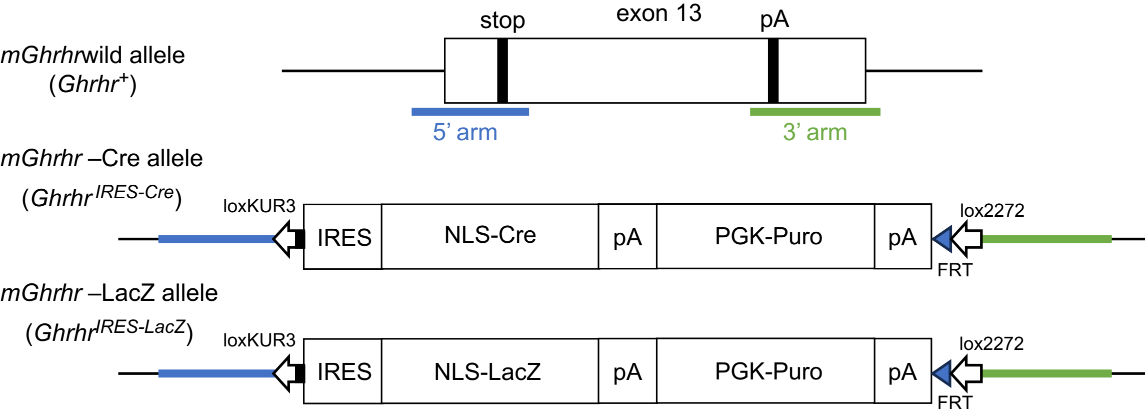

mGhrhr wild allele
(Ghrhr+)
stop
exon 13
pA
5' arm
3' arm
mGhrhr –Cre allele
(Ghrhr IRES-Cre)
loxKUR3
IRES
NLS-Cre
pA
PGK-Puro
pA
lox2272
FRT
mGhrhr –LacZ allele
(Ghrhr IRES-LacZ)
loxKUR3
IRES
NLS-LacZ
pA
PGK-Puro
pA
lox2272
FRT

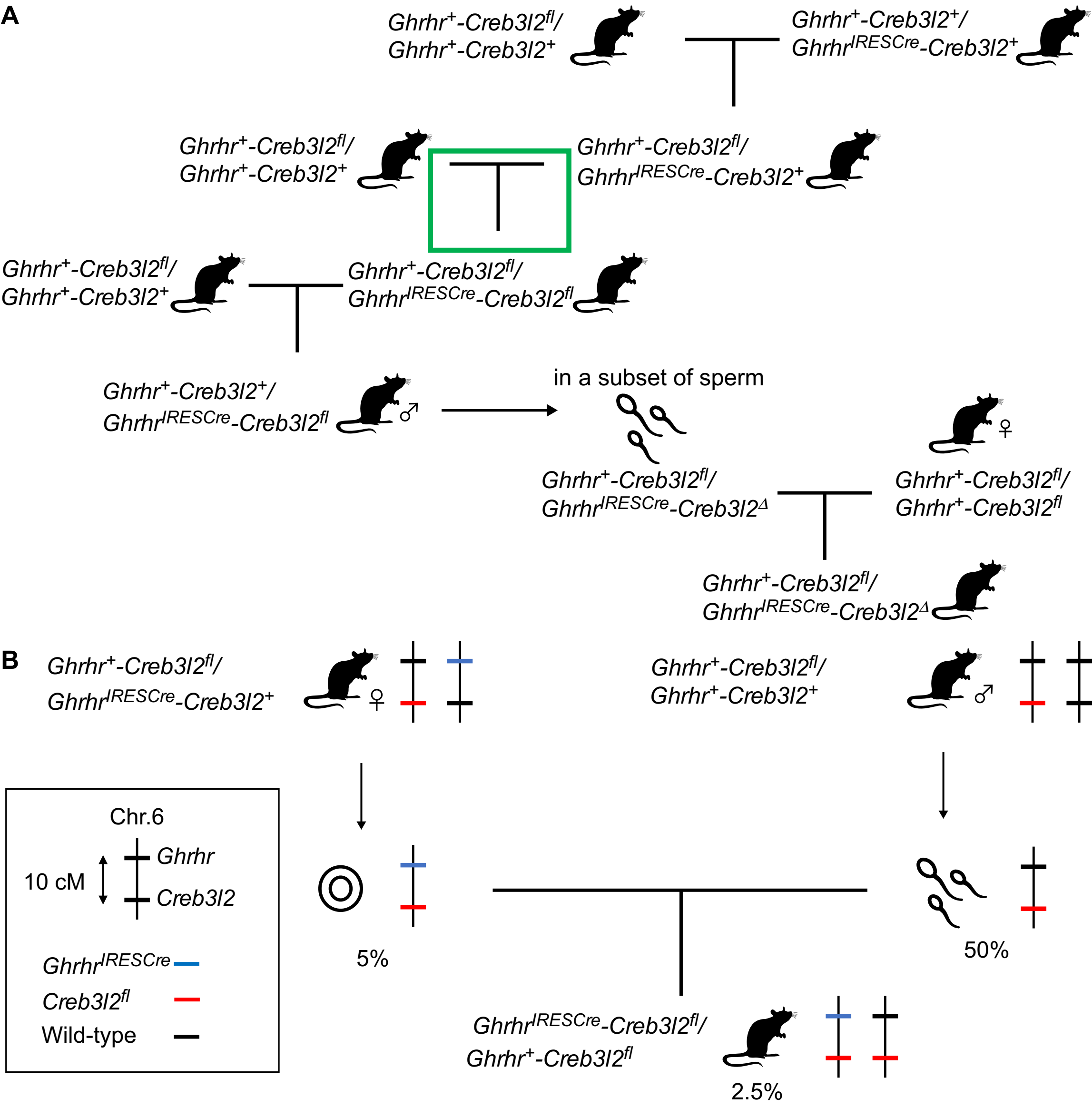

A
Ghrhr+-Creb3l2fl/
Ghrhr+-Creb3l2+
Ghrhr+-Creb3l2+/
GhrhrIRESCre-Creb3l2+
Ghrhr+-Creb3l2fl/
Ghrhr+-Creb3l2+
Ghrhr+-Creb3l2fl/
GhrhrIRESCre-Creb3l2+
Ghrhr+-Creb3l2fl/
Ghrhr+-Creb3l2+
Ghrhr+-Creb3l2fl/
GhrhrIRESCre-Creb3l2fl
Ghrhr+-Creb3l2+/
GhrhrIRESCre-Creb3l2fl ♂
in a subset of sperm
Ghrhr+-Creb3l2fl/
GhrhrIRESCre-Creb3l2Δ
♀
Ghrhr+-Creb3l2fl/
Ghrhr+-Creb3l2fl
Ghrhr+-Creb3l2fl/
GhrhrIRESCre-Creb3l2Δ
B
Ghrhr+-Creb3l2fl/
GhrhrIRESCre-Creb3l2+ ♀
Ghrhr+-Creb3l2fl/
Ghrhr+-Creb3l2+ ♂
Chr.6
10 cM
Ghrhr
Creb3l2
GhrhrIRESCre
Creb3l2fl
Wild-type
5%
50%
GhrhrIRESCre-Creb3l2fl/
Ghrhr+-Creb3l2fl
2.5%

Supplemental figure legends

Figure S1. Generation of *Ghrhr-Cre* driver mice and *Ghrhr-LacZ* mice.
In the last exon (exon 13) of the *Ghrhr* gene, a 5' arm containing a termination codon and a 3' arm containing a polyadenylation (pA) signal were designed. Through homologous recombination, mice were generated in which *NLS-Cre* or *NLS-LacZ* are transcribed in connection with *Ghrhr* mRNA. *loxKUR3* has a mutation in the right half of the *loxP* sequence, and *lox2272* has a mutation in the Cre-binding site, so there is no deletion between *loxKUR3* and *lox2272* by Cre. The tissue-specific expression of *Cre* and *LacZ* in each mouse line was confirmed by X-gal staining (data not shown).

Figure S2. **(A) Generation of pituitary somatotroph-specific Creb3l2 knockout mice.** To generate *Creb3l2* conditional knockout mice specific to somatotroph cells in the pituitary gland, we encountered an issue with the *Ghrhr* gene expression in the testes. When we used male *Ghrhr* $^{IRES\text{-}Cre/Creb3l2\ \mathrm{flox\ (fl)}}$ mice for mating, some of the sperm exhibited a deletion of exon 4 in the *Creb3l2*, resulting in the *Creb3l2Δ* allele. Consequently, offspring from these pairings displayed a systemic *Creb3l2* knockout. To avoid this, we adopted a strategy where we inherited the *Creb3l2 fl* allele from the female and both the *Creb3l2 Δ* allele and the *Ghrhr* $^{IRES\text{-}Cre}$ allele from the male, ultimately aiming to generate *Creb3l2* $^{fl/\Delta}$, *Ghrhr* $^{+/IRES\text{-}Cre}$ offspring.
**(B) Production of individuals carrying both *Ghrhr* $^{IRES\text{-}Cre}$ and *Creb3l2* $^{fl}$ alleles in cis.** To create individuals with both *Ghrhr* $^{IRES\text{-}Cre}$ and *Creb3l2* $^{fl}$ alleles on the same chromosome (in cis), we performed artificial insemination, mating male *Creb3l2* $^{+/fl}$, *Ghrhr* $^{+/+}$ mice with female *Creb3l2* $^{+/fl}$, *Ghrhr* $^{IRES\text{-}Cre/+}$ mice. This approach successfully produced *Creb3l2* $^{fl/fl}$, *Ghrhr* $^{+/IRES\text{-}Cre}$ male mice at an approximate probability of 2.5%.
